# How to use a multipurpose SNARE: The emerging role of Snap29 in cellular health

**DOI:** 10.15698/cst2018.04.130

**Published:** 2018-03-22

**Authors:** Valeria Mastrodonato, Elena Morelli, Thomas Vaccari

**Affiliations:** 1 Dipartimento di Bioscienze, Universita’ degli Studi di Milano, Italy.

**Keywords:** Snap29, membrane trafficking, endocytosis, autophagy, SNARE proteins, SNAP family, cell division

## Abstract

Despite extensive study, regulation of membrane trafficking is incompletely understood. In particular, the specific role of SNARE (Soluble NSF Attachment REceptor) proteins for distinct trafficking steps and their mechanism of action, beyond the core function in membrane fusion, are still elusive. Snap29 is a SNARE protein related to Snap25 that gathered a lot of attention in recent years. Here, we review the study of Snap29 and its emerging involvement in autophagy, a self eating process that is key to cell adaptation to changing environments, and in other trafficking pathways. We also discuss Snap29 role in synaptic transmission and in cell division, which might extend the repertoire of SNARE-mediated functions. Finally, we present evidence connecting Snap29 to human disease, highlighting the importance of Snap29 function in tissue development and homeostasis.

## INTRODUCTION

**Highlights:**
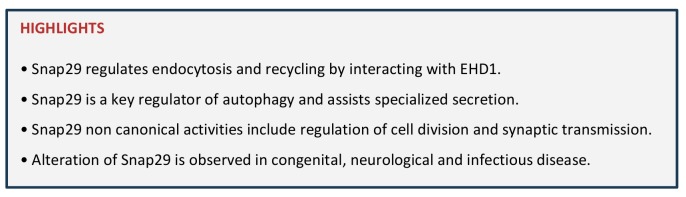


The specialized trafficking routes that evolved between compartments of the endomembrane system rely on a wide set of proteins, including primarily SNAREs, Rab GTPases and a large set of effectors and tethering factors, that ensure specificity of cargo delivery to a wide range of different target compartments. Precision and fidelity of targeting is paramount to support cell viability and to prevent disease. However, how such specificity is achieved at a molecular level is incompletely understood.

**Figure 1:**
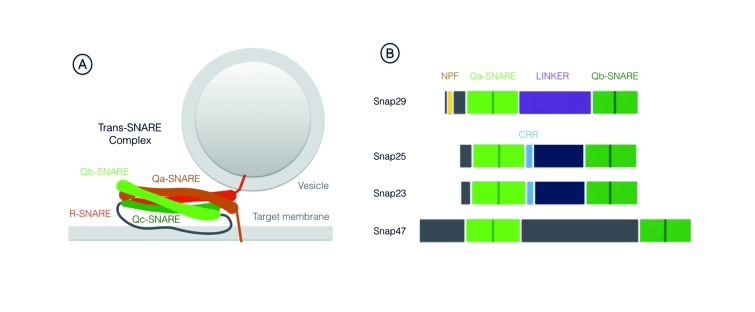
FIGURE 1: Illustration of the trans-SNARE complex involved in membrane fusion. All SNARE domains are oriented with the N-termini to the left **(A)**. Schematic representation of SNAP family members in mammals **(B)**. The homologous SNARE domains among the 4 paralogs are shown. The position of the central glutamine residue (Q) is indicated by a line. The linker region of Snap29 is (purple) differs from that of other family members and does not contain a cysteine rich region (CRR; light blue) for membrane targeting.The N-Terminal acidic NPF motif, exclusive to Snap29 among family members, is shown in yellow.

SNAREs are part of the conserved coiled-coil machinery that brings membranes in close proximity, a prerequisite for most membrane fusion events occurring during trafficking 1,2,3. A stereotypic set of SNARE proteins forming a 4-helix bundle (often referred to as trans-SNARE complex) composed of distinct SNARE domains named Qa-, Qb-, Qc- or R-SNARE are invariantly required for fusion. Usually, a Qa-SNARE-containing protein [often called syntaxin, or target (t)-SNARE] and a R-SNARE -containing protein [often called vesicle associated membrane protein (VAMP) protein, or v-SNARE] are carried by opposing membranes, each providing a SNARE domain to the fusion complex. These proteins are glued together by Qb- and Qc-SNARE domain containing proteins, providing the remaining 2 domains (**Fig. 1A**). After fusion, all proteins of the 4-helix bundle are associated to the target membrane, in an arrangement often called cis-SNARE complex, that is disassembled by the activity of a number of proteins including the ATPase NSF (N-ethylmaleimide-sensitive factor). The Qb- and Qc-SNAREs domains can also be contributed in a single protein, as is the case of members of the Synaptosomal-Associated Protein (SNAP) protein family. Snap25 and Snap23 are the most extensively studied SNAP family members (for review 4). Metazoan genomes further include Snap29, while the SNAP family in mammals comprises also Snap47 5,6. Here, we will focus on Snap29, whose functions have increasingly come into view in the last two decades.

Differently from other family members Snap29 possess an N-terminal acidic asparagine-proline-phenylalanine (NPF) motif. Similarly to Snap47, Snap29 also lacks cysteine residues in the spacer region between the two SNARE domains, that in Snap25 and Snap23 are modified to allow membrane anchoring 5 (**Fig. 1B**). Consistent with the lack of a membrane anchor, early biochemical studies revealed that Snap29 is only partially associated to membranes 5. They also suggested that Snap29 interacts with a wide range of syntaxins including Syntaxin6 (Syx6) a SNARE protein acting in the Golgi apparatus 5,7,8. Despite these initial studies, when compared to the advanced understanding of Snap25 and Snap23 function, the specificity and molecular regulation of Snap29 function remained to be explored. Below, we review the growing body of evidence indicating that Snap29 regulates membrane fusion at multiple cellular locales during intracellular trafficking. In addition, we report findings that indicate that Snap29 might not only be involved in membrane fusion, but that it could rather serve a regulatory or structural role in certain contexts. Finally, we discuss the involvement of Snap29 in human disease.

## ENDOCYTOSIS, RECYCLING AND CILIUM FORMATION REQUIRE SNAP29 AND ITS INTERACTOR EHD1 

**Figure 2:**
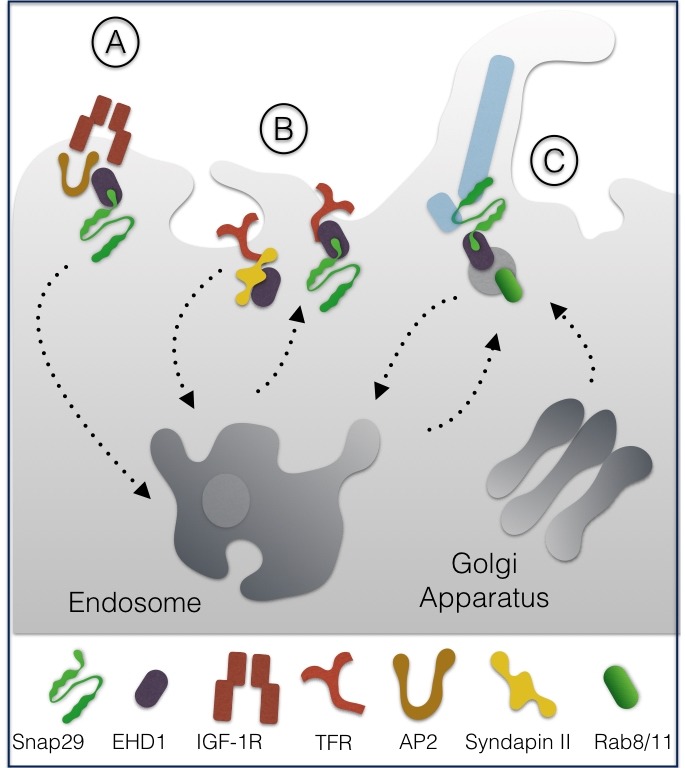
FIGURE 2: Roles of Snap29 in endocytosis and recycling. Snap29 is required for internalization of the IGF-1R receptor **(A)**, for internalization and/or recycling of TFR **(B)** and for formation of the basal membrane surrounding the cilium microtubules (light blue; **C**). All these functions are regulated in association with the endocytic factor EHD1, which binds the NPF motif of Snap29 (see text for details).

Together with the NPF interactor EHD1 and with the endocytic adapters AP2, Snap29 was reported to promote endosomal trafficking of the Insulin Growth Factor 1 receptor (IGF-1R) in CHO cells, a process crucial to down-regulate active receptors 9 (**Fig. 2A**).

Biochemical analyses showed that binding of EHD1 to the NPF motif of Snap29 can occur alternatively to that of the F-BAR protein syndapin II. Upon overexpression in HeLa cells, alternative EHD1 and syndapin II interaction to Snap29 affected internalization of transferrin receptors (TFR) but not of EGFR, suggesting that Snap29 might control trafficking of selected receptors 10 (**Fig. 2B**).

More recently, Snap29 has been shown to participate, both in RPE cells and in *Danio rerio*, in trafficking of ciliary vesicles, a process that depends on EHD1, on the recycling GTPase Rab11, and on the Golgi apparatus GTPase Rab8 11. The authors suggest that EHD1 is required for vesicle formation from preexisting membranes that fuse together and assemble in a membrane structure enveloping the cilium microtubules (MTs), by the action of Snap29-containing SNARE complexes (**Fig. 2C**). Interaction of Snap29 with EHD1 was also reported in *Drosophila melanogaster* 12.

## SNAP29 IS A KEY REGULATOR OF AUTOPHAGY

Macroautophagy (autophagy here after) is an adaptive cellular pathway operating in house-keeping as well as in stress conditions. It required for the degradation of damaged organelles, aggregated proteins and other potentially toxic waste material. Upon starvation, autophagy is also crucial for maintaining sufficient levels of cellular nutrients by self-digesting cytoplasmic molecules. Autophagic clearance requires the formation of an intermediate double-membrane organelle called autophagosome that fully engulfs the cargo before fusion with lysosome (for review 13).

In 2012, Itakura *et al*. reported that fusion of starvation-induced autophagosomes with lysosomes in HeLa cells requires Syntaxin17 (Syx17), Snap29 and the lysosomal R-SNARE Vamp8 14. In such work, the authors showed that Syx17 is recruited to autophagosomes from the endoplasmic reticulum (ER) and that Snap29 primarily associate to it, possibly from the cytoplasm. Subsequent characterization of Syx17 and Snap29 in *D. melanogaster *revealed a similar role in autophagy* in vivo *15,16.

The mechanism of Snap29-dependent regulation of autophagy was further dissected in human cells by showing that the binary Syx17-Snap29 complex on autophagosomes is stabilized by binding of ATG14 oligomers to Syx17. Such tethering function is proposed to be crucial for priming the complex for fusion with Vamp8 on the lysosomes 17.

**Figure 3:**
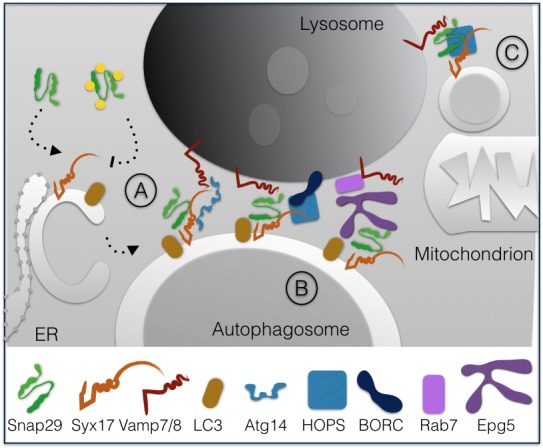
FIGURE 3: The function of Snap29 during autophagy. Snap29 associates to Syx17, which is recruited from the ER to nascent autophagosomes to regulate their fusion with lysosomes, with the help of Atg14 polymers. O-GlcNac modification of Snap29 in high nutrient conditions prevent inclusion in an fusion complex **(A)**. Fusion involves the HOPS and BORC tethering complexes, Epg5 and Rab7 **(B)**. Snap29, Syx17, Vamp8 and the HOPS complex are also used in fusion of mitochondrial-derived vesicles with lysosomes **(C)** (see text for details).

Interestingly, analysis of *Caenorhabditis elegans* mutants indicated that O-GlcNac modification of Snap29 is crucial to regulate autophagic clearance 18. In fact, in both worms and in human cells, it was found that in presence of high nutrients Snap29 is modified on 4 Serine residues, thus preventing inclusion in a functional SNARE complex. In contrast, in low nutrient conditions such as starvation, the unmodified Snap29 is readily included in the 4-helix bundle promoting autophagy and nutrient recovery (**Fig. 3A)**.

Fusion with lysosomes has been further shown to require the homotypic fusion and protein sorting (HOPS) tethering complex that associates with the late endosomal GTPase Rab7, both in *D. melanogaster* and human cells 19.

Fusion and HOPS recruitment is also assisted by the lysosome-associated multiprotein complex named BLOC-1 related complex (BORC) that regulates lysosome positioning 20. An additional factor controlling fusion of autophagosomes with lysosomes is Vici Syndrome Protein EPG5, a Rab7 effector that is found to interact with LC3/Lgg-1 in* C. elegans *and human cells to favor formation of the trans-SNARE complex 21. In absence of EPG5, inappropriate fusion of autophagosomes with early endocytic vesicles is shown to occur (**Fig. 3B)**.

Finally, recent data indicate that Snap29 acts in membrane fusion of Syx17-loaded mitochondrial derived vesicles to lysosomes with the help of the HOPS complex 22 (**Fig. 3C).**

## SNAP29 ASSISTS SPECIALIZED SECRETION

*In vivo *analyses of *C. elegans* and *D. melanogaster *lacking Snap29 revealed a wide range of trafficking defects, in addition to alteration of recycling, lysosomal degradation and blocked autophagy. These included a dispersed Golgi morphology, an impairment in Golgi trafficking and secretion, and, finally, inappropriate secretion of autophagosomes, suggesting that Snap29 acts directly during secretion 16,23,24. Consistent with this in a yeast two hybrid screen and in HeLa cells, it was found that SNAP29 interacts with the Golgi apparatus tethering factor COG6 and with the Golgi SNAREs Syntaxin5, SYX6 and GS27 25,26.

**Figure 4:**
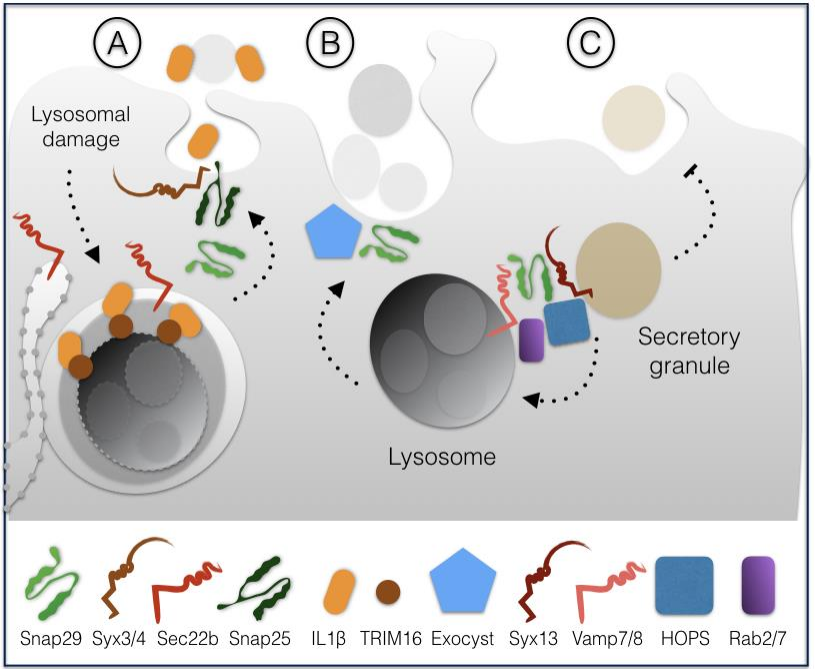
FIGURE 4: Snap29 activity in secretion. Release of the pro-inflammatory factor IL-1β requires fusion to the plasma membrane mediated by Snap29 **(A)**. Snap29 also regulates secretion of lysosomes during a developmental cell invasion process in *C.elegans ***(B)**. Regulation of secretion of glue granules in *D. melanogaster* salivary glands involves Snap29, which acts in fusion of excess granules to lysosomes **(C)** (see text for details).

In HeLa cells, Snap29 has also been directly involved in unconventional secretion of the leaderless pro-inflammatory factor interleukin-1β (IL-1β), which occurs upon lysosomal damage 27. The mode of IL-1β secretion is debated, but it has been proposed to involve formation of autophagosomes 28,29. Lysosomal damage can cause proteolytic cleavage activation of IL-1β, leading to binding to the receptor TRIM16 residing on the lysosomal membrane. In this way, IL-1β is sequestered within LC3 positive sequestration membranes, eventually forming an autophagosome 27. Depletion of Snap29, Snap23, Syntaxin 3 or 4 and of the ER-derived R-SNARE Sec22b affect IL-1β secretion, suggesting that autophagosomes containing IL-1β might fuse with the plasma membrane through the formation of a variety of SNARE complexes involving such SNAREs (**Fig. 4A**).

In *C. elegans* anchor cells, a developmental model of cell invasion that relies on secretion of lysosomes to the protruding front, depletion of Snap29 or of components of the exocyst, a complex involved in targeted exocytosis, has been found to inhibit protrusion formation 30. These data suggest that Snap29-mediated fusion might contribute to promote fusion of lysosomes to the plasma membrane in a highly specialized form of secretion (**Fig. 4B**).

A third specialized secretion pathway involving Snap29 has been recently identified in *D. melanogaster* 31. In pupal salivary glands, which secrete large amounts of granule containing glue, excess granules are cleared by lysosomes in a specialized form of autophagy termed crynophagy which was found to be lost upon depletion of Syx13, Snap29, Vamp7, the GTPase Rab2 and Rab7 and components of the HOPS tethering complex (**Fig. 4C**).

Finally, Snap29 is particularly abundant in non excitatory cells of the nervous system, such as rat oligodendrocytes in culture, especially during myelination 32. In this study, Snap29 was shown to colocalize and interact with the GTPase Rab3a via the N-terminal region of Snap29 and overexpression of Snap29 and Rab3a is reported to enhance cell surface trafficking of tagged myelin components, suggesting that Snap29 might regulate myelin secretion in glial cells 32. Considering that astrocytes and, in particular, microglia are involved in neuro-inflammation, it might be interesting to determine whether specific functions of Snap29 in these other types of glia cells exists.

## A NON TRAFFICKING ROLE OF SNAP29 POINTS TO A NEW MODEL OF OUTER KINETOCHORE FORMATION

**Figure 5:**
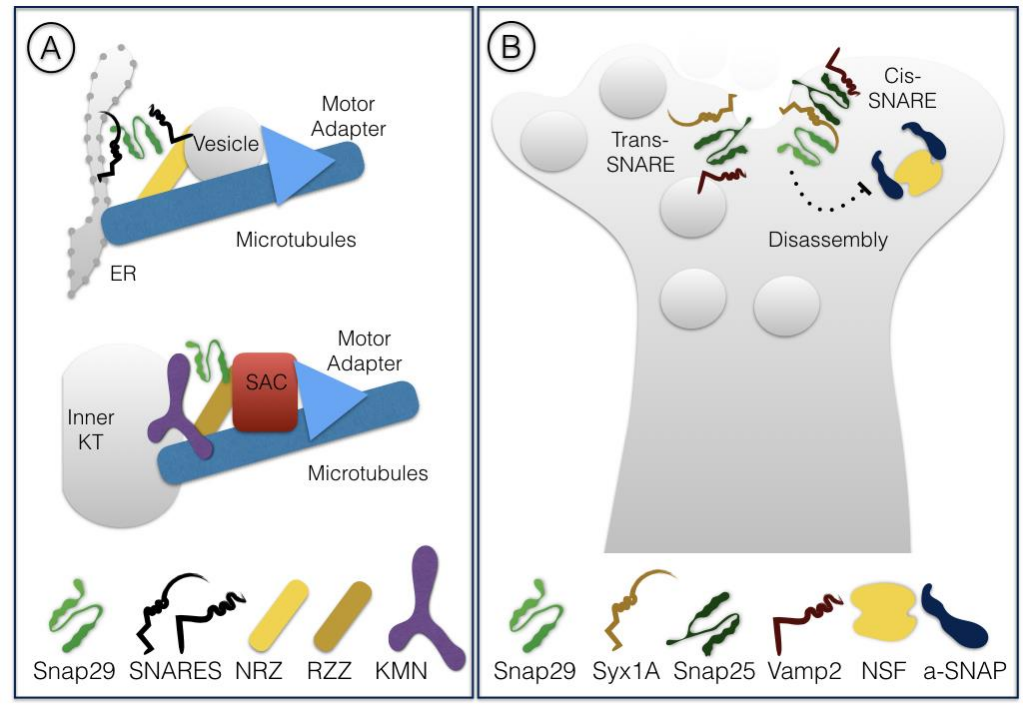
FIGURE 5: Non canonical roles of Snap29. Schematic representation of the function of Snap29 at synaptic vesicles **(A)** and at the kinetochore **(B)**. The comparison in A illustrates similarities between membrane trafficking and the membrane-independent process of kinetochore formation. The regulatory activity of SNAP29 during synaptic transmission illustrated in B is likely to involve binding to SNARE bundles containing SNAP25 (see text for details).

Surprisingly, we have recently found that during cell division Snap29 acts as a component of the kinetochore, the mitotic structure that connect condensed chromosomes to spindle microtubules 33. In *D. melanogaster* cells, we have observed relocalization of Snap29 to forming kinetochores at the onset of mitosis. Importantly, in both *D. melanogaster* and HeLa cells Snap29 depletion affects kinetochore formation and chromosome segregation. Electron microscopic analysis revealed that *D. melanogaster* Snap29 is present at the kinetochore in absence of membrane, suggesting that its association to the kinetochore might be independent of trafficking. Interestingly, RZZ, a well characterized kinetochore complex, shares components with the NRZ tethering complex that assists fusion into the ER of vesicles involved in retrograde trafficking of cargoes from the Golgi apparatus 34,35. Very recently, the structure of the RZZ complex has been resolved by cryo-electron microscopy and proposed to be similar of that of cytosolic coat scaffolds that mediate membrane trafficking in association with molecular motors and adapters 36. Thus, Snap29 might be part of a set of peripheral membrane proteins that are repurposed during cell division to mediate interaction of chromosomes with the microtubule cytoskeleton (**Fig. 5A**). While Snap29 has been found in studies of kinetochore proteins previously 37,38, further analysis is required to understand mechanistically the role of Snap29 at the kinetochore.

## SNAP29 FUNCTION IN THE NERVOUS SYSTEM: A NON CANONICAL MODE OF ACTION?

Snap29 was initially identified in immunoprecipitation experiments using rat brain extracts and in a yeast two-hybrid screen as an interactor of Syntaxin1A (Syx1A), which, together with Snap25, regulates synaptic transmission in neurons 39. Interestingly, Snap29 was found to be able to associate to a reconstituted SNARE complex consisting of Syx1A alone, Syx1A and Snap25, as well as the synaptic trimeric complex formed by Syx1A, Snap25 and Vamp2. In these experiments, binding of αSNAP, a factor necessary for disassembly of the SNARE complex after fusion to Syx1A complexes was weakened by the presence of Snap29, suggesting that Snap29 competes with αSNAP for binding to Syx1A complexes. However, Snap29 was not found to bind directly αSNAP, indicating that in presence of Snap29 disassembly of the SNARE complex is prevented and that Snap29 is likely to be included in the trimeric Syx1A, Snap25, Vamp2 complex. Injection of Snap29 in neurons in culture decreased the amplitude of synaptic firing, an effect that was reverted by coinjection of increasing amounts of αSNAP 39. In a subsequent study, the same group reported that hippocampal neurons depleted of Snap29 show increased efficiency of synaptic transmission 40. These studies suggest that Snap29 acts as a modulator of synaptic vesicle fusion, perhaps by inhibiting complex disassembly or substituting for Snap25 (**Fig 5B**).

*D. melanogaster* Snap29 also associates to the fly Syx1A homolog Syx1. The authors showed that Snap29 is not included in Snap25-containing cis-SNARE complexes recovered from fly mutants for the disassembly factor NSF, suggesting that Snap29 might be part of unstable SNARE complexes 12. In addition, Snap29 appears unable to rescue synaptic vesicle fusion in cultured Snap25-deficient neurons, suggesting that it is not functionally redundant with Snap25. In contrast, Snap29 is able to rescue, albeit very poorly, secretion of dense core vesicles containing neurotransmitters, which require prolonged neuron stimulation 41.

## SNAP29 AND DISEASE

Considering the wide involvement of Snap29 in multiple trafficking and non trafficking processes in diverse cell types, it is not surprising that alteration of Snap29 is associated to human disease (**Table 1**). Here, we review the consequences of alteration of human SNAP29 activity and we discuss potential mechanisms of pathogenesis.

**Table 1:** Table 1. List of diseases with proven alterations in SNAP29 (see text for details).

**Disease**	**Traits/Symptoms**	**SNAP29 involvement**	**Pathological mechanisms**	**References**
Schizophrenia	Cognitive and emotive dysfunction	Polymorphism in promoter region	Altered polarized transport in neurons?	42,43,44,45
CEDNIK	Congenital neuroectodermal defects	Loss of function mutations	Likely multiple	46,47,48,49,50,51
22q11.2DS	Mild to CEDNIK-like	Hemizigous mutations	Likely multiple	52
22q11.2 duplication (2 patients)	Ocular manifestations/Mild mental retardation and muscular hypotonia	Trisomy	Unknown	53,54
HPIV3 infection	Infantile brochiolitis and pneumonia	Binding of inhibitory Phosphoprotein P	Decreased autophagy	55
EV-A71 infection	Hand, foot, and mouth disease	Binding to VP0 and 2BC proteins	Increased autophagy	56

### Could schizophrenia involve an alteration of *SNAP29* gene activity?

An association of Snap29 with schizophrenia emerged in two early reports that revealed the presence of a polymorphism in the promoter region of *SNAP29* with schizophrenia patients 57,48. In addition, a study found the *SNAP29* promoter among the many bound by β-catenin, a transcription factor regulated by lithium, an antipsychotic drug 42, and a bioinformatic analysis associated *SNAP29* with a schizophrenia gene network 43. While alterations in *SNAP29* regulation in schizophrenia remain to be demonstrated, it is interesting to note that dysfunction of synaptic transmission has been proposed to be at the core of schizophrenia pathophysiology 44. Consistent with the possibility that regulation of *SNAP29 *gene expression might be altered in schizophrenia, SNAP29 has been found among the interactors of the schizophrenia susceptibility factor dysbindin, a component of the BLOC-1 complex, together with the Golgi adapter COG6 and the polarized transport molecules AP3, SEC6 and SEC8 in human neuroblast cells 45. Such evidence and that linking Snap29 to secretion and synaptic transmission (see previous chapter) suggest that Snap29 might act in trafficking processes subverted in schizophrenia.

### CEDNIK and other rare syndromes are caused by mutations in*SNAP29*

A more direct link of Snap29 with disease emerged in 2005. Indeed, it was reported that loss of SNAP29 cause CEDNIK (cerebral dysgenesis, neuropathy, ichthyosis, and keratoderma), a rare recessive congenital syndrome. In CEDNIK patients, homozygous *SNAP29* loss of function mutations cause a typical set of neurocutaneous traits that results in very poor life expectancy 46,47,48,49,50. The alterations due to lack of SNAP29 have been analyzed in the stratum corneum of skin of patients, which presents an accumulation of glucosylceramides normally secreted towards the upper layers, suggesting the existence of a defect in secretion of lamellar granules containing lipids and proteolytic enzymes important for normal skin development. Consistent with the role that SNAP29 plays in membrane trafficking, fibroblasts of CEDNIK patient possess aberrant morphology of the Golgi apparatus and of recycling endosomes, as well as defects in Golgi trafficking, endocytic recycling and cell motility, overall suggesting that alteration of these process might contribute to the pathogenesis of CEDNIK 51.

Patients affected by a 22q11.2 deletion syndrome (22q11.2DS), a relatively common micro-deletion of the region that includes the* SNAP29* gene, present a wide range of phenotypic abnormalities including immunodeficiency, palatal anomalies, congenital cardiovascular defects and additional symptoms varying from patient to patient. An exome study of 22q11.2 deletion syndrome patients revealed novel mutations in the remaining copy of *SNAP29*, suggesting that hemizygous mutations might unmask a recessive CEDNIK-like condition contributing to the phenotypic variety of patients 52. A similar scenario could apply to a patient affected by a uncommon form of 22q11.2DS 59, and to Di George syndrome, a rare multi-systemic condition also caused by heterozygous de novo deletions of the 22q11.2 region, which contains *SNAP29* and other 30-40 genes 60.

Finally, duplications in the region containing *SNAP29* have been found in 2 patients with congenital ocular, vascular and cranial nerve defects 53 and in 1 patient with mild facial dysmorphism and motor and intellectual delay 54. Overall, multiple congenital defects are associated to *SNAP29* alterations. However, given the pleiotropy of SNAP29 activity, the contribution of altered SNAP29 activity to the traits of these diseases appear complex and requires further examination.

### Infection is regulated by SNAP29 activity

The utmost importance of SNAP29 for regulation of fusion between autophagosomes and lysosomes is underscored by the finding that the capsid phosphoprotein P of human para influenza virus type 3 (HPIV3) is able to bind both SNAP29 SNARE domains. This interaction prevents binding of SNAP29 with SYX17, possibly preventing the formation of the ternary SNARE complex with VAMP8, required for autophagosome degradation. Since the accumulation of autophagosomes within the host cell is a prerequisite for virus particle release in the extracellular space, the study suggests that SNAP29 activity is a key factor to prevent HPIV3 infection 55.

The association of Snap29 with viral infection is not limited to HPIV3. In fact, a recent report showed that SNAP29 binds a structural protein of the enterovirus-A71 (EV-A71), which appear to up regulate autophagy for its replication 56. While the mechanism of SNAP29 regulation of EV-A71 is not yet clear, these two papers and the emerging notion that subversion of autophagy is a key step in the life cycle of viruses 61,62,63, indicates that SNAP29 might be a crucial factor to prevent virus infection.

The activity of SNAP29 to prevent infection is also potentially relevant to bacterial pathogens. Indeed, in mouse mast cells, an immune cell type specialized in clearing bacterial infections, Snap29 associates to *E. coli* containing phagosomes and its overexpression increases lysosomal clearance, suggesting that Snap29 assists antibacterial phagocytosis 64.

The pleiotropy of Snap29 activity and the massive increase in -*omics* approaches suggests that further association of Snap29 with common and rare disease might emerge. Considering the activities of Snap29 in processes associated to signal transduction, cell motility, cell division, autophagy and synaptic transmission, we predict an involvement in tumorigenesis and neurodegeneration.

## CONCLUSION

In the 20 years after Snap29 discovery, multiple studies have revealed a wide range of cellular processes controlled by the SNARE. Despite this, a number of outstanding question regarding the detailed mechanism of Snap29 remain unanswered. Here, we list some of the most outstanding.

1. As is the case of its paralog Snap23, Snap29 activity is not restricted a limited set of cellular locales. However, the mode of Snap29 targeting to membrane compartments appears radically different from that of Snap23 (and of Snap25), given the absence of the cysteine rich region and the charged amino acids that regulate membrane association 65. One factor that could regulate targeting of Snap29, at least during endocytic recycling is its interactor EHD1. However, how Snap29 is recruited to membranes during other trafficking processes remains to be understood.

2. So far the only reported post-translational modification of Snap29 is a O-GlcNac modification thought to regulate its activity during autophagy 18. Whether such modification affects other Snap29 functions is not understood and it remains to be determined whether other modifications might control Snap29 function.

3. How Snap29 acts in the SNARE complex is not entirely clear. While Snap29 appear to act positively in membrane fusion in multiple context, the inclusion of Snap29 in a 4-helix bundle has been demonstrated *in vitro* by X-ray crystallography only using its SNARE domains in separated form 17. The parallel orientation (N- to C- terminal) of each SNARE domain in the bundle posits that the linker region between the SNARE domains of Snap29 has to be disordered as that of Snap25 or Snap23. This possibility has not been yet tested.

4. Whether the 1st SNARE domain of Snap29 corresponds to a Qb and the 2nd to a Qc is also not clear and has been inferred mostly by analogy with Snap25 and Snap23. A large number of bundles employ 4 SNARE proteins, rather than 3, each carrying a SNARE domain. Thus, if the linker region of Snap29 was rigid enough to prevent inclusion of Snap29 in a single bundle, the paradigm of activity based on that of Snap25 would have to be revised. One interesting, albeit currently only theoretical, possibility is that Snap29 could tether multiple SNARE complexes by lending the 1st SNARE domain to a complex and the 2nd to the next. In this scenario, multiple identical bundles could be tethered without violating the Qa-, Qb-, Qc- R-SNARE rule of bundle formation.

5. Clarity on the molecular nature of the complexed formed by Snap29 will also aid to understand its role in synaptic transmission, which is likely relevant to the pathogenesis of CEDNIK and perhaps Schizophrenia. As explained above, Snap29 seems to play a regulatory role in neurons. Whether this is due to it being a competitor of Snap25 that is less efficient in membrane fusion, or whether Snap29 plays an inhibitory role in synaptic transmission remains to be determined. Interestingly, inhibitory SNAREs have been described 66 and the cytoplasmic localization of Snap29 could allow recruitment to SNAREs to regulate their availability to form a canonical bundle engaged in fusion. Both scenarios are consistent with the reported propensity of Snap29 to loosely associate to other SNAREs 12,67,68.

Remarkably, the yeast *S. cerevisiae *possesses two SNAP family members Sec9 and Spo20, the latter specifically required during meiosis to connect the prospore membrane to forming gametes during ascospore morphogenesis 69. Such event involves the enveloping of haploid nuclei by a double membrane, to form spores morphologically similar to autophagosomes. Similar to the case of Snap25 and Snap29, molecular dissection *in vitro *of Sec9 and Spo20 revealed that the former is a more active and more tightly bound to partner SNAREs 70. While the evolutionary relationships between the yeast and metazoan SNAP family members are not clear, it is tantalizing to speculate that Snap29 might be a metazoan evolutionary solution to a common eukaryotic necessity to diversify SNAP protein activity, to accommodate cell trafficking and, perhaps, non trafficking needs.

## Abbreviations

CEDNIK: cerebral dysgenesis, neuropathy, ichtyosis and keratoderma
HOPS: homotypic fusion and protein sorting
HPIV: human para influenza virus
IL: Interleukin
SNAP: synaptosomal-associated protein
SNARE: soluble N-ethylmaleimide-sensitive-factor attachment receptor
Syx: syntaxin
